# Necrosis of the Skull

**Published:** 1843-10

**Authors:** O. Hill

**Affiliations:** Detroit, Michigan


*Necrosis of the Skull.*—By O. Hill, M.D., Detroit, Michigan.—On the 13th of February last, whilst residing in Niagara county, New York, near the Falls, I was called to examine a female patient, aged about 45, laboring under extensive disease (necrosis) of the skull, of long standing. Her short history of the case was as follows:—About twenty-five years since she received an injury upon the left side of the head, from the falling of a stone from a building, which produced, as was then supposed, a slight fracture of the outer table, with severe concussion, rendering her insensible most of the time, for ten days. She recovered, however, under ordinary treatment without an operation, so far as to be able to go about her usual business. From that to the time of my first visit, has had at intervals, partial derangement of the mental faculties, confusion and want of consciousness of time and place; continuing sometimes for a few days, but remembering with accuracy all that happened up to these periods, and afterwards. Partial paralysis of the superior extremities of the side affected, and general wasting of the muscles and integuments. Small ulcers occasionally opening at different points in the scalp, discharging more or less sanious matter, which always relieved the confusion of ideas at the time. These openings would again close and remain so, sometimes from one to three years. About five years ago, as she says, "she was exposed to severe cold and froze the top of her head." Immediately after which, two large openings appeared, the size of a quarter of a dollar, one directly over, and within an inch of the left superciliary arch; the other an inch above the middle portion of the lambdoidal suture. From these ulcers a profuse discharge has constantly kept up for the last three years. On examination I found a small spicula of dead bone pointing at each of these ulcers. Seizing the two points with strong forceps, the whole left side of the skull seemed to be slightly moveable.

The constitution of the patient had become so enfeebled by the long-continued exhausting discharges, and her situation and circumstances not very conducive to recovery, I deemed any operation, at this late period hardly warrantable. However, as the patient and her friends were not only willing, but very anxious that something should be done, I resolved to undertake the removal of the dead bone, as the only remaining chance for her safety—as the system was fast sinking—and even this afforded but little encouragement, for it was impossible to foretell the nature and extent of the operation required, by an external examination. The inference was, that the powers of life must soon yield, and that any operation, however hazardous, would not materially hasten that period. This was the sensible reasoning of the patient.

The patient having already prepared herself, was placed on a seat, with the head inclined towards the *only window in the house*, and well supported by an assistant; I proceeded to divide the scalp, carrying the scalpel from one opening to the other, a distance of about seven inches, half an inch to the left of the median line, or sagittal suture. This at once exposed the whole extent of the bone, it being denuded mostly of its pericranium. After securing two or three branches of the occipital and temporal arteries, as they were much enlarged, the scalp was thrown off each way from the incision. On the left side the dissection was carried to within an inch of the squamous suture, before the outline of the disease could be determined in that direction. The left parietal bone was loose, and easily separated from its fellow, at the sagittal suture, so that the elevator could be introduced without the use of the trephine. Here a difficulty occurred in raising the dead bone, as it embraced all of the parietal, anterior to the parietal foramen, and more than half the left os frontis, so interlocked with spicula of new bone, that to throw out so large a piece, with its ragged edges, the elevator under one side only, would probably endanger the brain. I next proceeded to divide the dead portion with the cock's comb saw, near the line of the coronal suture, then by means of two or three elevators, raised and removed the frontal portion first, a partial line of demarcation having taken place. Here the saw also became necessary, as many points of new bone were found shooting and interlocking into the dead portion, requiring to be separated before it would yield to prudent force; then raising the sagittal edge of the parietal over its fellow, I drew it forcibly from its connection with the os temporum, at the squamous suture. This at once exposed the entire superior surface of the cerebrum of the left side, covered at rome points with pus and sanious matter, presenting the appearance at first view of one general diseased mass. The dura mater was wanting, as was also the pericranium—the disease having extended from the external to the internal surface, and throughout its whole extent.

Here many points of deep interest attracted our attention. On clearing away the matter, the brain, with its pia mater in perfect order, was seen packed away, as if intelligently avoiding injury, occupying the lower part of the cavity, leaving a space above between it and the bone, of an inch and a half, or more, and by its violent pulsations gave abundant evidence that it was still striving to perform its functions. The longitudinal sinus was seen enclosed in its fold of the dura mater, having been detached by the ulcerative process from the dead bone, but adhering firmly to the healthy side. I do not mean to be understood that the whole of the parietal bone was removed. A line drawn from the parietal foramen to the highest point of the squamous portion of the temporal bone, would be about the line of demarcation. Thus avoiding most wisely, the principal branches of the great meningeal artery—leaving, of course, the temporal and occipital angle of the bone sound. The patient was calm and collected during the whole operation, which occupied nearly half an hour, answering questions intelligently, except for a moment when the brain was first exposed; and when attempts were made to clear the surface, she seemed drowsy, complained of deafness, and inclined to faint, but soon roused by the use of diffusible stimulants and wine.

The wound was cleansed, scalp replaced, and secured by sutures and straps, and the patient put to bed—feeling, as she expressed herself, much better than she had done for years before. Previous to the operation for a long time, she had been afflicted at times with extreme pain down the left side of the neck, extending to the left lung and stomach, occasionally cramp of the left arm and muscles of the neck, twitching of the left eye, &c. All these difficulties ceased the same day of the operation, and have not returned up to this time. The patient was immediately put upon a liberal nutritious diet of animal food and porter, and every attention paid to her comfort. No unusual degree of inflammation arose. The scalp healed kindly by the first intention, osseous matter soon began to be deposited, and up to this time (six months since the operation) has continued to do well. Bony arches are shooting over at many points, which, with a slight approximation of the sides of the opening, is fast forming a substantial covering. A small opening was left for a few weeks, at the anterior axtremity for the exit of matter, through which the brain could be seen gradually expanding, and apparently resuming its functions. The patient walks and rides, is cheerful and intelligent, and bids fair to repair the breach yet to a very considerable extent.

Perhaps it may not be unworthy of remark, that the facts in this case, go to establish a physiological *fact* of vast practical importance, that the human system, physically and mentally, is made up of two distinct halves or parts, that their functions are not necessarily connected, but may, and do, go on independently. I say of vast importance, in a practical point of view, because it teaches us that lesions of structures, and even a total loss of portions of the most important organs, do *not* stop the machinery, or necessarily prove fatal to the animal. Hence we might often be induced to resort to remedial measures, and as often be astonished at our success, in many cases that are now abandoned to their fate, through the timidity of the practitioner, to act with boldness and decision. As in the foregoing case, one half of the cerebrum was packed away, its healthy functions suspended, and therefore useless, if not destroyed, as was evinced in the corresponding deviations from healthy action in remote parts depending upon it for their sensations and motions, if not for their vitality. Yet the nutritive, respiratory, digestive, and and all other functions, the senses, &c., were carried on, as it appeared, for years with sufficient force and regularity, for all ordinary purposes. The other animal, or the other half, must have been in a comparatively normal condition, to have sustained all these functions alone, *so* perfectly, and no direct connection, nervous or otherwise, could have existed between the parts diseased, and the corresponding healthy parts. This case may also serve to establish the notion of the insensibility of the upper surface of the brain; at all events experiments of this sort, upon the living organ itself, is the only way to arrive at any conclusion on this point. In this case the effects upon the nervous system did not seem to arise from irritation and disease of the surface of the brain, but from pressure by the pent up matter, and thickening of parts. The brain was handled, sponged, and thoroughly cleansed, without any evidences of pain in the part, or extremities—the patient only appearing a little dull, from pressure of the sponge. Besides, all the unpleasant symptoms, before mentioned, as indicating disturbance of the nervous system, subsided immediately on removing all pressure, leaving no traces of irritation or inflammation. I forbear any further comments.—*Boston Medical Journal*, Sept. 13, 1843.

